# Computer Vision in Human Analysis: From Face and Body to Clothes

**DOI:** 10.3390/s23125378

**Published:** 2023-06-06

**Authors:** Mohamed Daoudi, Roberto Vezzani, Guido Borghi, Claudio Ferrari, Marcella Cornia, Federico Becattini, Andrea Pilzer

**Affiliations:** 1 CNRS, Centrale Lille, Institut Mines-Télécom, UMR 9189 CRIStAL, University of Lille, F-59000 Lille, France; mohamed.daoudi@imt-lille-douai.fr; 2 IMT Nord Europe, Institut Mines-Télécom, Centre for Digital Systems, F-59000 Lille, France; 3 Department of Engineering “Enzo Ferrari”, University of Modena and Reggio Emilia, 41100 Modena, Italy; roberto.vezzani@unimore.it; 4 Department of Computer Science and Engineering, University of Bologna, 47521 Cesena, Italy; 5 Department of Engineering and Architecture, University of Parma, 43121 Parma, Italy; claudio.ferrari2@unipr.it; 6 Department of Education and Humanities, University of Modena and Reggio Emilia, 42121 Reggio Emilia, Italy; marcella.cornia@unimore.it; 7 Media Integration and Communication Center, University of Florence, 50100 Florence, Italy; federico.becattini@unifi.it; 8 Dipartimento di Ingegneria dell’Informazione e Scienze Matematiche, University of Siena, 53100 Siena, Italy; 9 Department of Computer Science, Aalto University, 02130 Espoo, Finland; andrea.pilzer@aalto.fi

## 1. Introduction

For decades, researchers of different areas, ranging from artificial intelligence to computer vision, have intensively investigated human-centered data, i.e., data in which the human plays a significant role, acquired through a non-invasive approach, such as cameras. This interest has been largely supported by the highly informative nature of this kind of data, which provides a variety of information with which it is possible to understand many aspects including, for instance, the human body or the outward appearance. Some of the main tasks related to human analysis are focused on the body (e.g., human pose estimation and anthropocentric measurement estimation), the hands (e.g., gesture detection and recognition), the head (e.g., head pose estimation), or the face (e.g., emotion and expression recognition). Additional tasks are based on non-corporal elements, such as motion (e.g., action recognition and human behavior understanding) and clothes (e.g., garment-based virtual try-on and attribute recognition). Unfortunately, privacy issues severely limit the usage and the diffusion of this kind of data, making the exploitation of learning approaches challenging. In particular, privacy issues behind the acquisition and the use of human-centered data must be addressed by public and private institutions and companies.

Thirteen high-quality papers have been published in this Special Issue and are summarized in the following: four of them are focused on the human face (facial geometry, facial landmark detection, and emotion recognition), two on eye image analysis (eye status classification and 3D gaze estimation), five on the body (pose estimation, conversational gesture analysis, and action recognition), and two on the outward appearance (transferring clothing styles and fashion-oriented image captioning). These numbers confirm the high interest in human-centered data and, in particular, the variety of real-world applications that it is possible to develop.

## 2. Overview of Contribution

The human body represents one of the most investigated elements in the literature and in our Special Issue. In [1], the authors propose a system that can predict the future skeleton sequence through the integration of the surrounding situation directly into the presented model. In particular, the accuracy is improved for motions related to humans and objects. Amadi et al. [2] analyze the segmentation of human body parts through the usage of optimized 2D poses, validating the approach on the *Transportation Security Administration Passenger Screening Dataset* (TSA-PSD). The task of 3D human pose estimation is addressed in [3], in which the authors propose the use of bidirectional gated recurrent units to predict the global motion sequence from the local pose sequence. Gestures are investigated in [4], where a method for capturing gestures automatically from videos and transforming them into stored 3D representations is proposed. In [5], the authors exploit body joints to predict action progress.

Another topic investigated in this Special Issue is human face analysis. In particular, the published papers address different topics, including the problem of machine interaction using voice commands and facial movements [6], 3D face and body geometry reconstruction [7,8], and dyadic interaction analysis based on facial expressions [9]. Focusing on eye images, Gibertoni et al. [10] propose a system to automatically classify the eye status in images acquired through an ophthalmic tool. The authors suggest that this solution can help to improve the quality of future datasets acquired in this field, also simplifying the operations of non-technical figures, such as doctors. The second work concerning the human eye is described in [11] and consists of a framework developed to identify the user’s attention in a corneal imaging system. The proposed system is based on infrared and RGB images and, through an eyeball model, a final prediction of the 3D direction of the gaze is output.

Finally, two papers focus on the problem of outward appearance and fashion. In particular, Fontanini et al. [12] propose a method for transferring clothing styles across images of people, while Moratelli et al. [13] propose an image captioning approach for fashion retrieval applications.

## 3. Conclusions

The main goal of this Special Issue is to improve the communication between companies and researchers belonging to both private and public institutions regarding the opportunities (and limitations) of the use of human-centered data in the development of future artificial intelligence applications. The above-mentioned papers contribute to stimulating new ideas, motivations, and methodologies that can shape the future of this area, also outlining potential future industrial applications and prospective trends. Again, we remark on the importance of the proper use of data concerning humans, which must be compliant with privacy and ethical regulations.
